# Research on Bell-Shaped Vibratory Angular Rate Gyro's Character of Resonator

**DOI:** 10.3390/s130404724

**Published:** 2013-04-10

**Authors:** Zhong Su, Mengyin Fu, Qing Li, Ning Liu, Hong Liu

**Affiliations:** 1 Institute of Intelligent Control, Beijing Information Science & Technological University, Beijing 100101, China; E-Mails: liqing@bistu.edu.cn (Q.L.); liuning1898@bit.edu.cn (N.L.); kalmanliuhong@126.com (H.L.); 2 School of Automation, Beijing Institute of Technology, Beijing 100084, China; E-Mail: fumy@bit.edu.cn

**Keywords:** bell-shaped vibratory angular rate gyro, Coriolis vibratory gyro, bell-shaped resonator

## Abstract

Bell-shaped vibratory angular rate gyro (abbreviated as BVG) is a new type Coriolis vibratory gyro that was inspired by Chinese traditional clocks. The resonator fuses based on a variable thickness axisymmetric multicurved surface shell. Its characteristics can directly influence the performance of BVG. The BVG structure not only has capabilities of bearing high overload, high impact and, compared with the tuning fork, vibrating beam, shell and a comb structure, but also a higher frequency to overcome the influence of the disturbance of the exterior environment than the same sized hemispherical resonator gyroscope (HRG) and the traditional cylinder vibratory gyroscope. It can be widely applied in high dynamic low precision angular rate measurement occasions. The main work is as follows: the issue mainly analyzes the structure and basic principle, and investigates the bell-shaped resonator's mathematical model. The reasonable structural parameters are obtained from finite element analysis and an intelligent platform. Using the current solid vibration gyro theory analyzes the structural characteristics and principles of BVG. The bell-shaped resonator is simplified as a paraboloid of the revolution mechanical model, which has a fixed closed end and a free opened end. It obtains the natural frequency and vibration modes based on the theory of elasticity. The structural parameters are obtained from the orthogonal method by the research on the structural parameters of the resonator analysis. It obtains the modal analysis, stress analysis and impact analysis with the chosen parameters. Finally, using the turntable experiment verifies the gyro effect of the BVG.

## 1. Introduction

A solid-state wave gyroscope can be used to measure the angular velocity of a rotating body based on the inertia effect of the standing wave in two vibration modes of the axisymmetric resonator, which have advantages, such as small size, high operation accuracy, low cost, low power consumption, good shock resistance, and long life [1,2]. Axisymmetric vibratory structures with piezoelectric, magnetic or electrostatic actuators are widely used in vibratory gyroscopes, such as hemispherical resonator gyros (HRG), cylindrical resonant gyros (CRG), disk resonant gyros (DRG) and so on [3]. These structures are always activated and sensed by methods, including electromagnetics, electrostatics and piezoelectricity [4]. Symmetric vibratory structures with piezoelectric, magnetic or electrostatic actuators are widely used in vibratory gyroscopes, such as hemispherical resonator gyros (HRG), cylindrical resonant gyros (CRG), disk resonant gyros (DRG) and so on [3]. These structures are always activated and sensed by methods, including electromagnetics, electrostatics and piezoelectricity [4].

The hemispherical resonator gyroscope (HRG) was developed rapidly in Delco, Litton, and Northrop Grumman Co. [5], which has achieved inertial navigation performance levels and been used for spacecraft stabilization, precision pointing, aircraft navigation and strategic accuracy systems. Innalabs Holding manufactured the Coriolis vibratory gyroscope (CVG) with a metallic cylindrical resonator (tactical grade) [6], and Watson industries designed the vibrating structure gyroscope (VSG) with a piezoceramic cupped structure (rate grade) [7]. The Innalabs CVG and Watson VSG are typical cylindrical solid-state wave gyroscopes, which are suitable for land vehicle control, avionics systems, missiles and naval equipment. In this article, we explore the possibility of developing a novel vibratory gyroscope based on the piezoelectric effect and present the design, analysis, simulation and experiment of the novel gyroscope, which is named the bell-shaped vibratory angular rate gyro (abbreviated as BVG). This one is a new type of Coriolis vibratory gyro that is inspired by Chinese traditional clocks. The resonator fuses are based on a variable thickness axisymmetric multi-curved surface shell. Its characteristics will directly influence the performance of BVG. The BVG structure not only has capabilities of bearing high overload, high impact and, compared with the tuning fork, vibrating beam, shell and a comb structure, but also has a frequency to overcome the influence of the disturbance of the exterior environment, compared with the hemispherical resonator gyroscope (HRG) and the traditional cylinder vibratory gyroscope. It is most important that the Chinese traditional clocks have more stability, that the sound spreads fast and heavy, that the vibration time is long and that they bear high overload. The BVG is inspired by Chinese traditional clocks. The BVG's innovation consists of four aspects:

1. The gyroscope is a simple millimeter-scale metallic gyro, which is comprised of a resonator, piezoelectric elements and a capacitor. It is easier to manufacture and fabricate than that with micro-machining technology;
2. The bell-shaped resonator can bear a higher impact than others;
3. The accuracy is improved using capacitor-detecting technology;
4. It can be widely applied in the high dynamic low precision angular rate measurement occasions.

## 2. Design Principle Description

**Figure 1.**
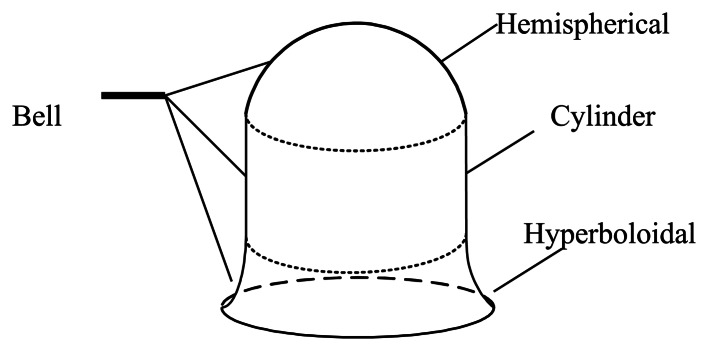
Traditional bell.

**Figure 2.**
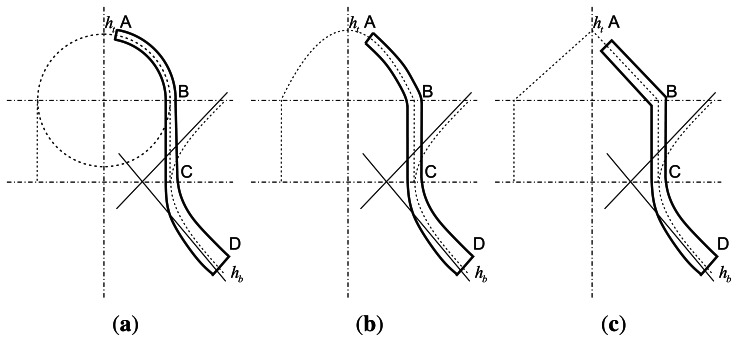
Cross-section of bell-shaped resonator with a variable thickness axisymmetric multi-curve.

**Figure 3.**
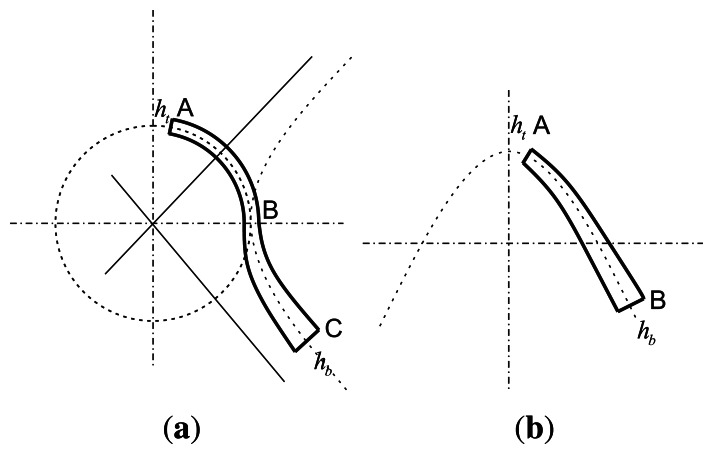
Cross-section of a bell-shaped resonator with a variable thickness axisymmetric multi-curve.

The schematic sketch of the traditional bell is shown in Figure 1. As far as we know, the bell includes a hemispherical shell, a cylinder shell and a hyperboloid shell. Figure 2 shows the cross-section of an open bell-shaped shell element with a variable thickness axisymmetric multi-curved surface. The shell is used for the sensitive element of the BVG, which is named the bell-shaped resonator. The meridian of the bell-shaped resonator has a hyperboloid ended (CD) at the waist circle and is connected to an open cylinder (BC), then connected to an open hemispherical (AB). In order to obtain the character, one can evaluate the character for this bell-shaped resonator with linearly varying thickness by means of synthesizing them for each part [8]. It is difficult to describe in one mathematic function. We want to find one function to describe the meridian of the bell and research the character of the resonator. The bell-shaped resonator is simplified by the two curves (see Figure 3(a)). Figure 3(a) shows the cross-section of a simplified resonator. In the initial process of researching the character of the resonator, the bell-shaped resonator is simplified to the paraboloidal shell (see Figure 3(b)). The paraboloid is the easiest bell-shaped resonator. The character of the paraboloid is analyzed in this article, so as to lay the foundations for the bell-shaped resonator with a variable thickness axisymmetric multi-curved surface shell.

### 2.1. The Structure of the Gyroscope

**Figure 4.**
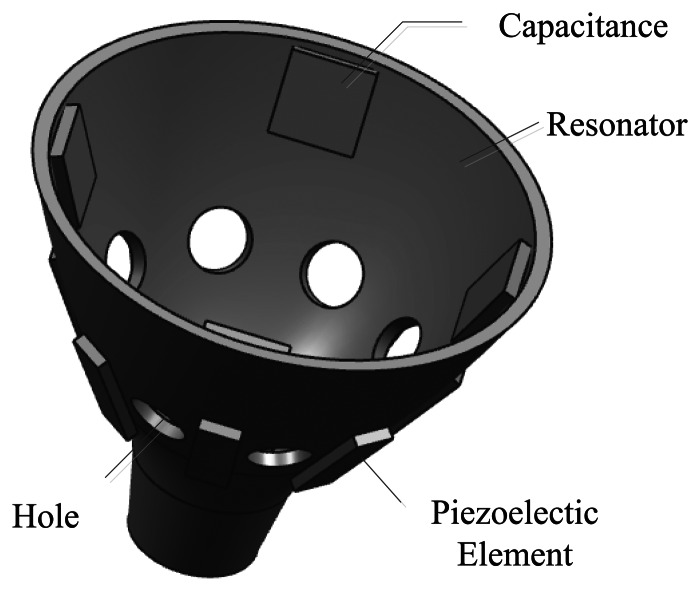
Schematic of a bell-shaped resonator.

The schematic sketch of the presented bell-shaped resonator is shown in the figure (see Figure 4). The sensor has a simple structure, comprised of a resonator, eight piezoelectric elements and four capacitor plates. The active and sense control piezoelectric elements are attached to the outer surface of the resonator. The sense control capacitor plates are attached to the inner surface. The overall structure is symmetrical. There are eight holes between the piezoelectric element to isolate the vibration of the one next to it.

### 2.2. Gyro Working Principle

**Figure 5.**
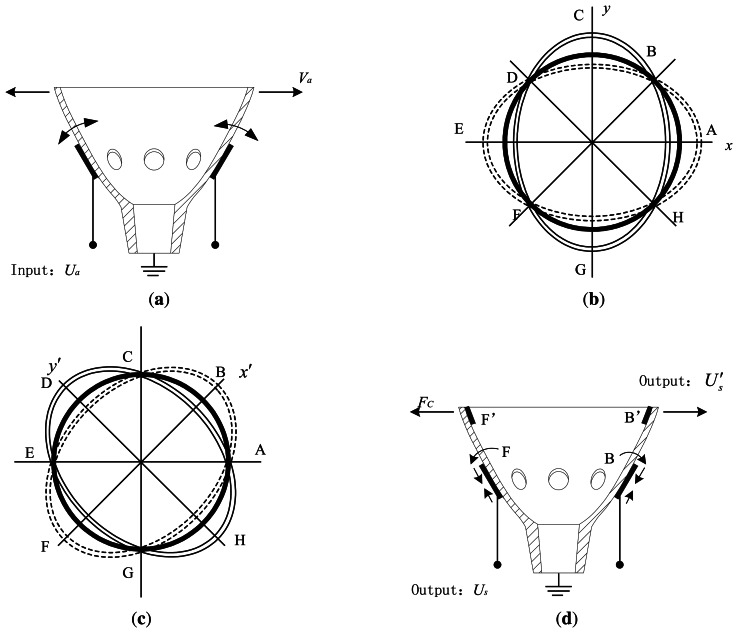
Schematic of the working principle.

The working principle of the BVG is based on the inertia effect of the standing wave in two vibration modes of the axisymmetric resonator caused by the Coriolis force. The schematic diagram of the working principle is shown in Figure 5. For the converse-piezoelectric effect, the piezoelectric elements contract and expand, which produce alternating bending moments on surface. By applying alternate voltage to the excitation elements (piezoelectric elements A and E, shown in Figure 5(a)), a standing wave is created on the bottom edge (the active mode, shown in Figure 5(b)), which has four nodes and antinodes.

In this case, if the gyroscope is rotating about the symmetry axis with an angular velocity, Ω (to measured), the Coriolis force, *F_c_*, in the resonator, which is perpendicular the vibration velocity vector of the active mode and the angular velocity vector, excites the bottom edge into another circle-ellipse flexural vibration in the *x′* − *y′* direction (the sense mode, shown in Figure 5(c)). In the sense mode, the resonator in *x′* − *y′* axes vibrates, and the piezoelectric elements (B, F, D and H) attached to it contract and ex0pand; alternatively, for the piezoelectric effect, the strain of piezoelectric elements produce an output signal, *U_s_* (shown in Figure 5(d)). Meanwhile, the vibration causes the change of the distance between the pair of capacitor plates (B′, F′, D′ and H′) and changes the value of the capacitor, Δ*C*. *U_s_* and Δ*C* are proportional to angular velocity, Ω, and can be detected by arithmetic and a readout circuit.

## 3. Modeling and Analysis

**Figure 6.**
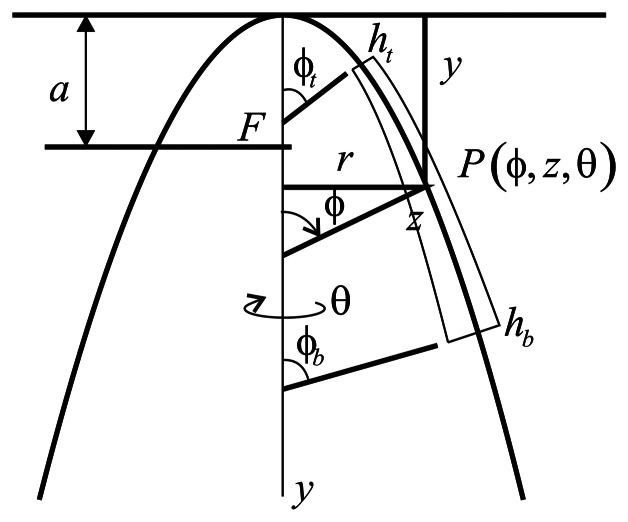
Cross-section of an open paraboloidal resonator with variable thickness.

The paraboloidal resonator and coordinate system are shown in Figure 6, which is essentially that given by Leissa *et al.* [9].

The resonator's middle surface is generated by rotating the meridian line about the *y*-axis, which is a parabola, *y* = *r*^2^/4*a*, where *a* is the focal distance. The thickness (*h*) of the resonator varies in the meridional direction, *ϕ*. The thicknesses at the top (*ϕ* = *ϕ_t_*) and at the bottom (*ϕ* = *ϕ_b_*) ends of the resonator are denoted by *h_t_* and *h_b_*, respectively Thus, the resonator is generated by rotating the cross-section of Figure 6 one revolution about the *y*-axis (0 ≤ *θ* ≤ 2*π*). The typical point, *P*, in the resonator is located by giving its meridional and circumferential angles, *θ* and *ϕ*, respectively, and by the distance, *z*, measured along the normal plane, measured from the midsurface. Thus, the resonator surface is at *z* = ±*h*/2. The ends of the resonator are located by the meridional angles, *ϕ_t_* and *ϕ_b_*, as shown in Figure 6.

The two principal radii of curvature for the midsurface of the paraboloidal shell are:

(1) $$\rho_{1}=\frac{2a}{\cos^{3}\phi },\;\rho_{2}=\frac{2a}{\cos\phi }$$

Where *ρ*_1_ describes the curvature of the midsurface in the meridional (*r* − *y*) plane and *ρ*_2_ is in a plane normal to the meridian. The latter is the distance along the normal plane from the middle surface to the axis of rotation (*y*), as seen in Figure 5(a).

Generating equations of the motion of the paraboloidal body (Ω = 0) are [10]:

(2) $$\{\begin{array}{l} \sigma_{\phi z,z}+\frac{1}{r_{z}}[\sigma_{\phi \theta ,\theta }+\sigma_{\phi z}\sin\phi +(\sigma_{\phi \phi }-\sigma_{\theta \theta })\cos\phi ]+\frac{1}{\rho_{z}}(\sigma_{\phi \phi ,\phi }+2\sigma_{\phi z})+f_{\phi }=\rho {\ddot{u}}_{\phi }\\ \sigma_{zz,z}+\frac{1}{r_{z}}[\sigma_{z\theta ,\theta }+(\sigma_{zz}-\sigma_{\phi \phi })\sin\phi +\sigma_{\phi z}\cos\phi ]+\frac{1}{\rho_{z}}(\sigma_{\phi z,\phi }-\sigma_{\phi \phi }+\sigma_{zz})+f_{z}=\rho {\ddot{u}}_{z}\\ \sigma_{z\theta ,z}+\frac{1}{r_{z}}(\sigma_{\theta \theta ,\theta }+2\sigma_{z\theta }\sin\phi +2\sigma_{\phi \theta }\cos\phi )+\frac{1}{\rho_{z}}(\sigma_{\phi \theta ,\phi }+\sigma_{z\theta })+f_{\theta }=\rho {\ddot{u}}_{\theta } \end{array}$$

where *υ_ϕ_*, *υ_z_* and *υ_θ_* (circumferential) are displacement components, *ρ_z_* = *ρ*_1_ + *z* and *r_z_* = (*ρ*_2_ + *z*) sin *ϕ* and *ρ* is mass density per unit volume.

Assuming a linearly elastic, isotropic material, the stress-strain equations are:

(3) $$\begin{array}{ccc} \sigma_{\phi \phi }=\lambda \varepsilon +2G\varepsilon_{\phi \phi }, & \sigma_{zz}=\lambda \varepsilon +2G\varepsilon_{zz}, & \sigma_{\theta \theta }=\lambda \varepsilon +2G\varepsilon_{\theta \theta }\\ \sigma_{\phi z}=2G\varepsilon_{\phi z}, & \sigma_{\phi \theta }=2G\varepsilon_{\phi \theta }, & \sigma_{z\theta }=2G\varepsilon_{z\theta } \end{array}$$

where *λ* and *G* are the Lam coefficients:

$$\begin{array}{cc} \lambda =\frac{E\mu }{\left(1+\mu )(1-2\mu \right)}, & G=\frac{E}{2(1+\mu )} \end{array}$$

and *ε* ≡ *ε_ϕϕ_* + *ε_zz_* + *ε_θθ_*.

The strain-displacement equations are found to be:

(4) $$\begin{array}{l} \varepsilon_{\phi \phi }=\frac{1}{\rho_{z}}(u_{\phi ,\phi }+u_{z})\\ \varepsilon_{zz}=u_{z,z}\\ \varepsilon_{\theta \theta }=\frac{1}{r_{z}}(u_{\theta ,\theta }+u_{\phi }\cos\phi +u_{z}\sin\phi )\\ \varepsilon_{\phi z}=\frac{1}{2}\left[u_{\phi ,z}-\frac{1}{\rho_{z}}(u_{\phi }-u_{z,\phi })\right]\\ \varepsilon_{\phi \theta }=\frac{1}{2}\left[\frac{1}{r_{z}}(u_{\phi ,\theta }-u_{\theta }\cos\phi )+\frac{u_{\theta ,\phi }}{\rho_{z}}\right]\\ \varepsilon_{z\theta }=\frac{1}{2}\left[\frac{1}{r_{z}}(u_{z,\theta }-u_{\theta }\cos\phi )+u_{\theta ,\phi }\right] \end{array}$$

In the present work, paraboloidal shells of revolution are analyzed by a 3-D approach. Instead of attempting to solve equations of motion, an energy approach is followed, which, as sufficient freedom is given to the three displacement components, yields frequency values as close to the exact ones as desired.

An elastic body undergoing free, undamped vibration has potential energy and kinetic energy. The potential energy (*V*) is the strain energy due to deformation. This may be expressed in terms of stresses *σ_ij_* and strains *ε_ij_* as:

(5) $$V=\frac{1}{2}\int_{\Omega }(\sigma_{\phi \phi }\varepsilon_{\phi \phi }+\sigma_{\phi \phi }\varepsilon_{\phi \phi }+\sigma_{\phi \phi }\varepsilon_{\phi \phi }+2\sigma_{\phi z}\varepsilon_{\phi z}+2\sigma_{\phi \theta }\varepsilon_{\phi \theta }+2\sigma_{z\theta }\varepsilon_{z\theta })\rho_{z}r_{z}d\phi d_{z}d\theta$$

The kinetic energy of the thick shell is:

(6) $$T=\frac{1}{2}\int_{\Omega }\rho \;({\dot{u}}_{\phi }^{2}+{\dot{u}}_{z}^{2}+{\dot{u}}_{\theta }^{2})\rho_{z}r_{z}d\phi \text{dzd}\theta$$

For the free, undamped vibration, the time (*t*) response of the three displacements is sinusoidal, and moreover, the circular symmetry of the body of revolution allows the displacements to be expressed by:

(7) $$\begin{array}{l} u_{\phi }(\phi ,z,t)=U_{\phi }\;(\phi ,z)\cos n\theta \sin(\omega t+\alpha )\\ u_{z}(\phi ,z,t)=U_{z}\;(\phi ,z)\cos n\theta \sin(\omega t+\alpha )\\ u_{\theta }(\phi ,z,t)=U_{\theta }\;(\phi ,z)\sin n\theta \sin(\omega t+\alpha ) \end{array}$$

where *n* is an integer (0, 1,…, ∞), *ω* is the natural frequency and *α* is a phase angle, depending upon the initial conditions. The solution of the equation have been given by Leissa *et al.* [8,9,11,12].

## 4. Simulation

In this paper, our work is simulated by FEM (finite element method) software, which could solve the modal frequency, the modal displacements distribution, the electric displacement distribution due to response displacements and the couple field analysis.

### 4.1. Impact Dynamics Analysis

**Figure 7.**
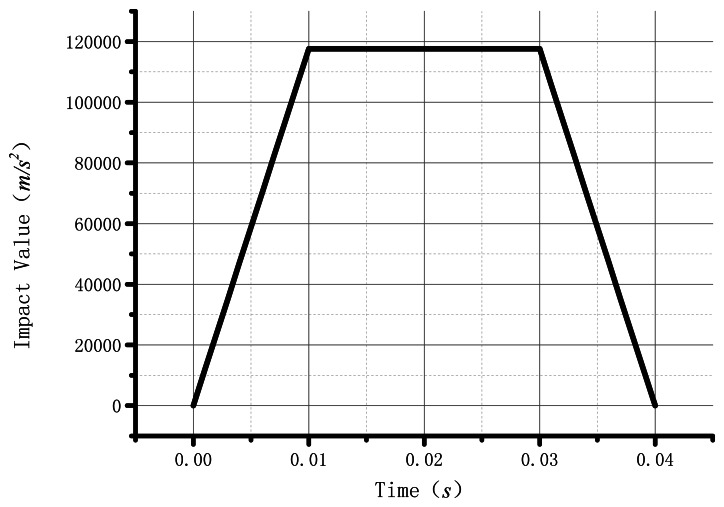
The diagram of impact.

The benefit of BVG is the ability to load a higher impact, which can adapt well to the high dynamic environment. The impact is the system's sudden change of force, displacement, speed and acceleration in transient excitation. The FEM could help us in analyzing how the resonator changes in the impact process. The impact value, corresponding to the time in transient excitation, is shown in Figure 7. Table 1 gives the parameters the the analysis used.

**Figure 8.**
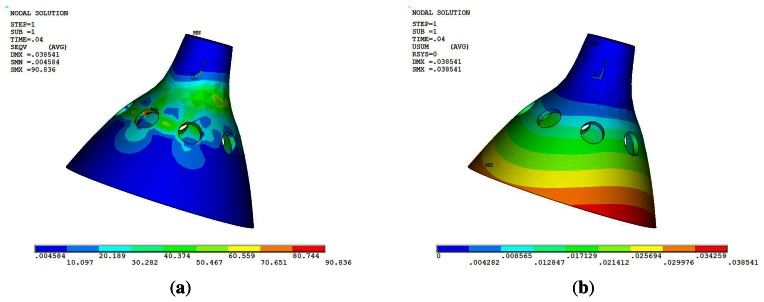
Schematic of the working principle.

The maximum stress disappears at 10 ms during the impact process, corresponding to the Von Mises stress distribution and the displacements distribution, as shown in Figure 8.

Figure 8 shows that the maximum Von Mises stress at 90.836 Mpa is much smaller than the yield strength. It is verified that the resonator does not occur in the plastic deformation during the impact process. The maximum displacement is 0.038 mm.

### 4.2. Modal Analysis

**Figure 9.**
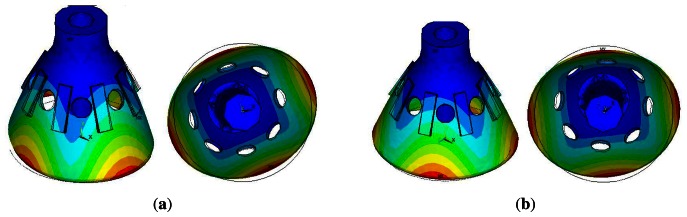

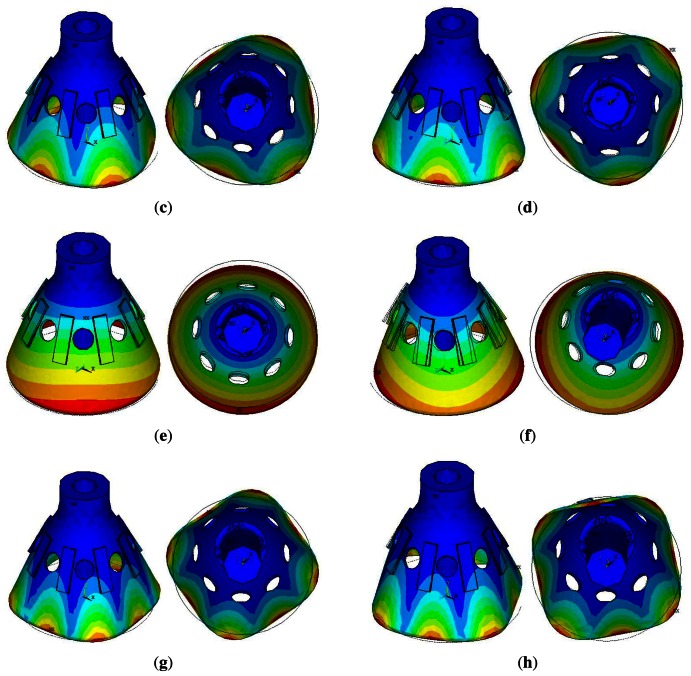
The result of mode analysis.

In order to get the appropriate modal frequency and modal shape, the dimensions of the entire structure are optimized in the modal analysis module. The modal analysis results are shown in Figure 9. The active mode frequency is 5,909.6 Hz, which is coincident with the sense mode. The equivalent frequency of the active mode and sense mode enables the Coriolis force to induce the sense mode of high sensitivity. The equivalent frequency of the active mode and sense mode enables the Corio-lis force to induce a sense mode of a high sensitivity [13]. The other order of mode frequency is shown in Table 2.

According to the modal contour of the FEM model, we can find the active mode and sense mode of the resonator. In fact, there are some calculation errors and grid errors that influence the frequency.

### 4.3. Parameter Selection

In fact, there are other physical factors and geometrical factors that influence the sensitivity of the BVG, such as the Young's modulus, piezoelectric constants, natural frequency and other dimension parameters. The influence of the main structure parameters on static and dynamic characteristics is investigated comprehensively by using an orthogonal test design. The steps of the orthogonal test are as follows.

**Figure 10.**
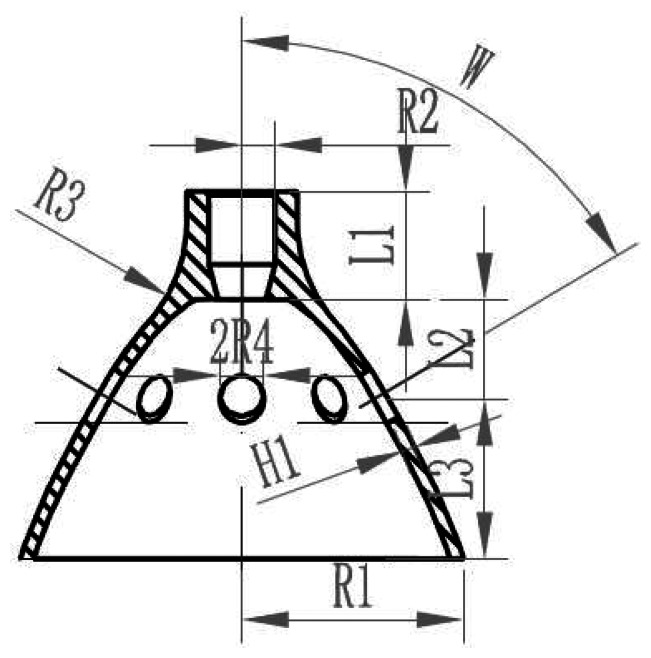
The schematic diagram of the resonator structure.

Step1: Confirm the experiment factor on the target index.
  - The active mode and sense mode frequency of the resonator (target: 6, 000*Hz*−7, 000*Hz*);
  - the difference between the previous mode frequency with the active mode (target: > 1, 500*Hz*);
  - the difference between the follow mode frequency with the sense mode (target: > 1, 500*Hz*).
Step2: Confirm the experiment factor (see Figure 10).
  - The top cylinder length (L1)
  - The radius of the resonator (R1)
  - The thickness of the resonator (H1)
  - The length between the top and the isolation hole (L2)
  - The length between the isolation hole and the bottom edge (L3)
Step3: Confirm the factor level of the test design.
Step4: Confirm the orthogonal table. (see Table 3)
Step5: Confirm the headers of the Table 4.
Step6: Program the test plan and experiment based on Table 3 and 4.
Step7: Results analysis.
Step8: Next test.

This method got the optimal parameter after the experiment as follows: H1 = 0.7 mm; R1 = 11 mm; L3 = 9 mm; L2 = 6 mm; L1 = 6 mm.

## 5. Experiment and Analysis

### 5.1. Fabrication

The metallic resonator of the prototypal gyroscope is made out of a Cu-based alloy doped with Ni and Cr. In order to obtain high elasticity, a high Q-factor, and homogeneous density of the metallic resonator, some thermal treatment procedures, such as solid solution and age strengthening, are carefully programmed before precision machining.

**Figure 11.**
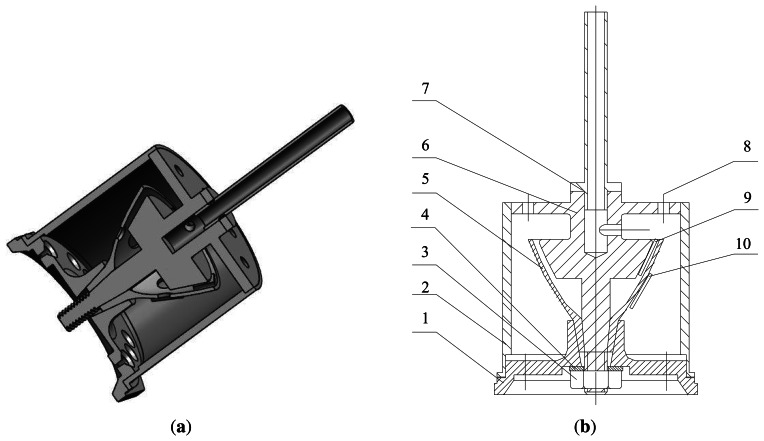
BVG sensor assembly unit: (**a**) 3-D sensor unit; (**b**) schematic sketch.

Figure 11 shows the assembly unit of the BVG. The core of the BVG includes: 1. The base of the resonator; 2. The outer cover of the resonator; 3. Nut; 4. Shim; 5. The bell-shaped resonator; 6. The central axis; 7. Air exhaust; 8. Wiring terminal; 9. Capacitor; 10. Piezoelectric elements

### 5.2. Modal Test

**Figure 12.**
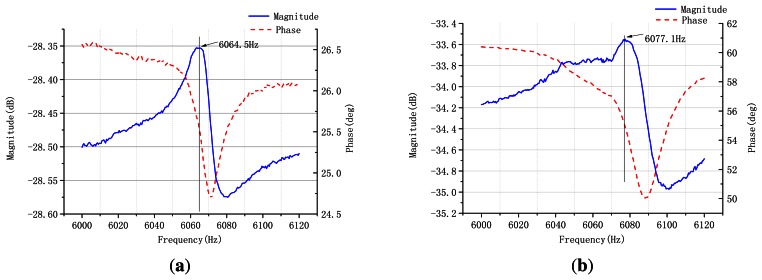
Resonating response of active mode: (**a**) active mode; (**b**) sense mode.

A modal test is used to validate the vibration of the sensor's work modes. The harmonic response is tested using a TD1250-C frequency response analyzer. In active mode test, the inducing voltage is applied on piezoelectric elements A and E, and the response voltage is picked up from piezoelectric elements C and G; the inducing voltage is applied on piezoelectric elements B and F, and the response voltage is picked from piezoelectric elements D and H. The resonating response is shown in Figure 12.

From the response curves of the active mode and sense mode, the gyroscope resonates at the frequency of 6,064.5 Hz and 6,077.1 Hz, approximately, which is close to the FEM simulation result. The frequency split of two work modes is 12.6 Hz. Using the method to restrain the frequency split, we use the method in [14].

### 5.3. Mode Shape Test

**Figure 13.**
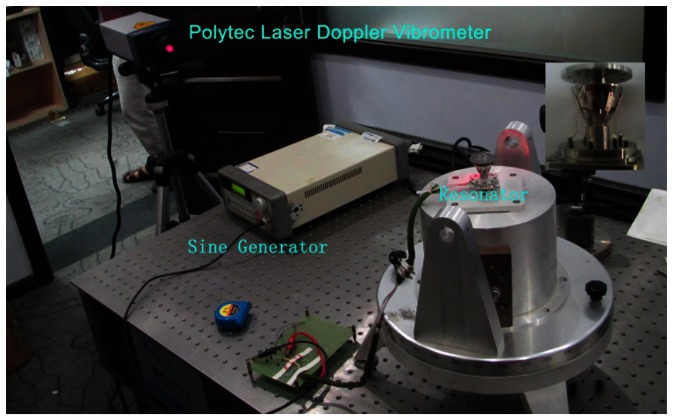
The resonator test.

To verify the resonator mode shape, we use the Polytec PSV-400 scanning vibrometer to test the radial displacement amplitude (see Figure 13).

**Figure 14.**
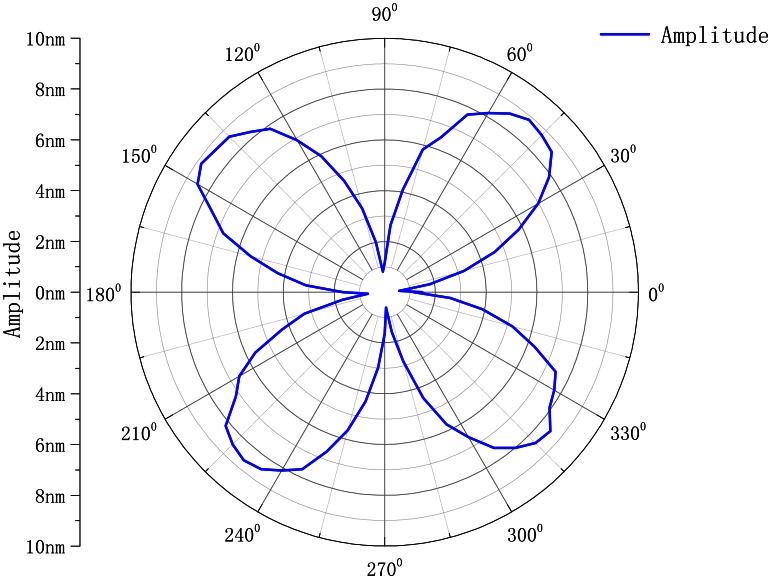
Mode shape of the resonator.

The circumference of the resonator was divided into 72 parts. The active frequency is 6,064.5 Hz; the amplitude is 5 V. We get the curve as shown in Figure 14.

As shown, the circumference of the resonator produces the four-wave mode shape. However, it is not the standard mode shape. The reasons for the bias are as follows:

- The influence of piezoelectricity;
- The errors of the signal generator;
- The noise of the experiment circuit;
- The position of the piezoelectric element on the surface of the resonator;
- The manufacture errors of the resonator.

### 5.4. Control Circuit

**Figure 15.**
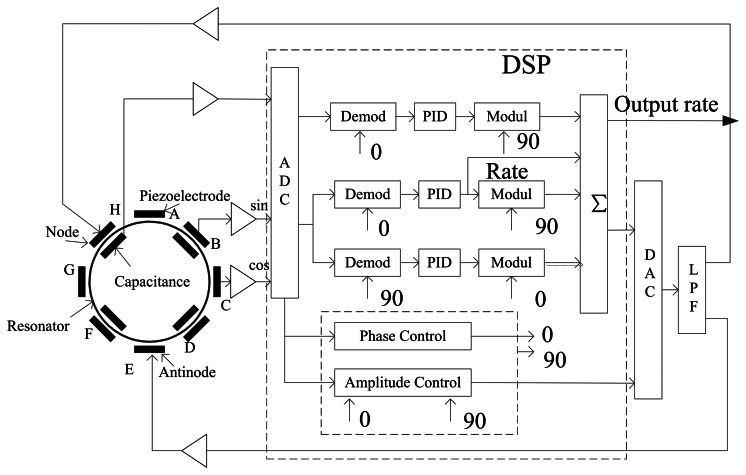
The block diagram of the control method and the photo of the control circuit.

The control circuit is very important in the overall performance of the BVG. It is designed on the classical vibratory gyroscope, and the diagram is shown in Figure 15.

### 5.5. Gyroscopic Effect

**Figure 16.**
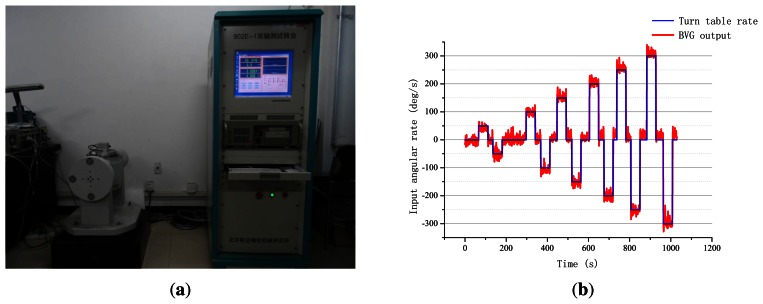
The turntable test.

The variable angular rate was applied to a single axis of a turntable to verify that BVG produced the gyroscopic effect. The test results are given in Figure 16. The BVG is a real gyroscope, just as the traditional.

## 6. BVG Future Opportunities

Although a lot of effort is being spent on improving these weaknesses, an efficient and effective method has yet to be developed. Next, it is important that we research the “Real” bell-shaped resonator, as shown in Figure 2(a), using the method of analysis for the paraboloidal resonator.

## 7. Conclusions

This article presents the modeling, simulation and fabrication of BVG. BVG based on the piezoelectric effect of active and capacitor detection has a solid metallic structure on the millimeter scale, which can be manufactured by conventional machining technology. Compared with the conventional vibratory gyroscope, the novel structure could load a higher impact. It can be widely applied in high dynamic low precision angular rate measurement occasions. It obtained reasonable structural parameters with the finite element analysis and an intelligent platform. The main work is as follows: the issue mainly analyzes the structure and basic principle and investigates the bell-shaped resonator's mathematical model. Using the current solid vibration gyro theory analyzes the structural characteristics and principles of BVG. The bell-shaped resonator is simplified as a paraboloid of the revolution mechanical model, which has a fixed closed end and a free opened end. It obtains the natural frequency and vibration modes based on the theory of elasticity. The structural parameters are obtained from the orthogonal method by research on the structural parameters of the resonator analysis. It obtains the modal analysis, stress analysis and impact analysis with the chosen parameters. Finally, using the turntable experiment verifies the gyro effect of the BVG. However, the quality factor and performances of this gyro are not so attractive now compared to other similar gyros. The main reason are as follows: the influence of material temperature character, the frequency split of the resonator, the algorithm of the control loop and so on. Next, the important work is as follow: restrain the frequency split, compensate for the temperature, research the advanced algorithm about the signal of the gyro and so on.

## Figures and Tables

**Table 1.** The parameters with simulation.

**Name of the Parameters (Unit)**	**Value**
Material	Ni43CrTi (3J53)
Density (*kg*/*m*^3^)	8170
Poisson's ratio	0.3
Young modulus (*GPa*)	196.76
Yield strength (*MPa*)	500
Simulation method	Transient dynamic
Mesh generation method	Free
Release ratio	1*E* − 4
Piezoelectric element	PZT-5A

**Table 2.** The parameters with simulation.

**The Order of Mode**	**Frequency Value (Hz)**
First	5909.3
Second	5914.6
Third	8924.8
Fourth	8929.2
Fifth	10,478.6
Sixth	10,479.2
Seventh	15,603.7
Eighth	15,626.8

**Table 3.** *L*^16^(2^15^) orthogonal table.

No.	1	2	3	4	5	6	7	8	9	10	11	12	13	14	15
1	(1)	3	2	5	4	7	6	9	8	11	10	13	12	15	14
2		(2)	1	6	7	4	5	10	11	8	9	14	15	12	13
3			(3)	7	6	5	4	11	10	9	8	15	14	13	12
4				(4)	1	2	3	12	13	14	15	8	9	10	11
5					(5)	3	2	13	12	15	14	9	8	11	10
6						(6)	1	14	15	12	13	10	11	8	9
7							(7)	15	14	13	12	11	10	9	8
8								(8)	1	2	3	4	5	6	7
9									(9)	3	2	5	4	7	6
10										(10)	1	6	7	4	5
11											(11)	7	6	5	4
12												(12)	1	2	3
13													(13)	3	2
14														(14)	1
15															(15)

**Table 4.** Headers of the table.

Factors	L1	H1		R1				L2							L3
Column	A	B	A×B	C	A×C	B×C	D×E	D						A×E	E
